# An automatic speech analytics program for digital assessment of stress burden and psychosocial health

**DOI:** 10.1038/s44184-023-00036-9

**Published:** 2023-09-13

**Authors:** Amanda M. Y. Chu, Benson S. Y. Lam, Jenny T. Y. Tsang, Agnes Tiwari, Helina Yuk, Jacky N. L. Chan, Mike K. P. So

**Affiliations:** 1 Department of Social Sciences and Policy Studies, The Education University of Hong Kong, Tai Po, Hong Kong, China; 2 Department of Mathematics, Statistics and Insurance, The Hang Seng University of Hong Kong, Shatin, Hong Kong, China; 3 School of Nursing, Tung Wah College, Ho Man Tin, Hong Kong, China; 4 School of Nursing, The University of Hong Kong, Pokfulam, Hong Kong, China; 5 School of Nursing, Hong Kong Sanatorium & Hospital, Happy Valley, Hong Kong, China; 6 Department of Social Work, The Chinese University of Hong Kong, Shatin, Hong Kong, China; 7 Department of Information Systems, Business Statistics and Operations Management, The Hong Kong University of Science and Technology, Clear Water Bay, Hong Kong, China

**Keywords:** Public health, Quality of life

## Abstract

The stress burden generated from family caregiving makes caregivers particularly prone to developing psychosocial health issues; however, with early diagnosis and intervention, disease progression and long-term disability can be prevented. We developed an automatic speech analytics program (ASAP) for the detection of psychosocial health issues based on clients’ speech. One hundred Cantonese-speaking family caregivers were recruited with the results suggesting that the ASAP can identify family caregivers with low or high stress burden levels with an accuracy rate of 72%. The findings indicate that digital health technology can be used to assist in the psychosocial health assessment. While the conventional method requires rigorous assessments by specialists with multiple rounds of questioning, the ASAP can provide a cost-effective and immediate initial assessment to identify high levels of stress among family caregivers so they can be referred to social workers and healthcare professionals for further assessments and treatments.

## Introduction

Psychosocial wellness, which encompasses mental, emotional, social, and spiritual well-being, is an essential component of health. However, psychosocial health issues are increasingly recognized as a public health crisis and a global economic burden. According to the World Health Organization, there has been a 13% rise in mental and substance use disorders in the last decade, that is, between 2007 and 2017^1^. Mental disorders cause 1 in 5 years lived with disability. Between 1990 and 2019, the global number of disability-adjusted life years lost due to mental disorders increased from 80.8 million to 125.3 million^2^. The direct and indirect costs of psychosocial health issues have amounted to over 4% of the global GDP. These costs totaled more than the combined cost of cancer, diabetes, and chronic respiratory diseases^3^. The Global Burden of Disease 2019 showed that psychosocial health issues were among the top ten leading causes of disease burden around the globe^2^.

These issues were markedly intensified by the COVID-19 pandemic. Being one of the biggest global crises in recent generations, the COVID-19 pandemic has had long-lasting and far-reaching repercussions for health systems, economies, and societies. Countless people have been infected and many of those are still suffering from long COVID. Businesses have gone bankrupt and millions of people have fallen below the poverty line. Children and young people have missed out on precious learning and socializing opportunities^4^. It has, therefore, created enormous stress among the global population. The number of people reporting symptoms of stress-induced anxiety and depression has profoundly increased by 30% amid the pandemic^5^. In 2021, the World Health Organization announced that depression has become a leading cause of disability worldwide^6^. Psychosocial health issues resulting from acute panic, anxiety, obsessive behaviors, hoarding, paranoia, and depression served as major contributors to the overall global burden of disease^7^.

Family caregivers are particularly prone to developing psychosocial health issues. According to the Family Caregiver Alliance^8^, all unpaid individuals who assist in the activities of daily living for other family members are family caregivers. In the context of an aging population, lengthening life expectancy, increasing chronic illnesses, and diversifying family structures, the number of family caregivers is on the rise^9,10^. Caregiving activities create additional physical, psychosocial, and financial burdens for caregivers, thereby leading to stress^11^. Previous reports have indicated that high levels of stress burden are related to the psychosocial health issues of family caregivers, particularly those with low family resilience^12^. Family resilience refers to effective coping strategies and adaptation in the face of losses, hardships, or adversities. It involves the ability to get through crises successfully and become stronger as a result. The capability of a family to manage and move forward from a disruptive experience will influence both the immediate and long-term adaptation of the entire family unit and thus is closely correlated with the caregiver’s stress burden^13^. The challenges of caregiving have been exacerbated by the COVID-19 pandemic; family caregivers reported increased duties and stress burden together with a decrease in family resilience, thereby resulting in psychosocial problems^14,15^.

The early detection of, and intervention in, psychosocial health issues can prevent disease progression and lessen long-term disability^16,17^. The underlying diseases of psychosocial health issues can be effectively treated at an early stage and at a relatively low cost. Primary healthcare, therefore, plays an important role in the management of these issues. Moreover, identifying individuals with psychosocial health issues—specifically those belonging to vulnerable groups, such as family caregivers—for targeted support is a public health priority.

Although it is emphasized that psychosocial health is a fundamental human right and is essential to the development of all countries^2^, only five out of ten people that require psychosocial health care services can access the care they need in high-income countries. In low- and middle-income countries, this number is even lower, constituting one out of ten people^18,19^. The cumbersome and subjective assessment process used to identify psychosocial health issues is a major obstacle to eradicating these issues. Detailed individual interviews are typically considered the gold standard of psychosocial health assessments. This conventional method involves rigorous assessments by specialists based on validated instruments such as questionnaires. It contains multiple rounds of questioning for a wide range of symptoms of mental health problems and social adjustment disorders and therefore, it may take up to a couple of hours to complete^20^. If interviews are recorded and need to be manually transcribed into text before data processing, it will take even longer and require more human resources. These time- and labor-consuming assessments impede the early detection of, and intervention in, psychosocial problems. Moreover, the assessments derived from clients’ responses in the interviews can be subjective; for example, owing to the stigma of psychosocial problems, clients may find it embarrassing to openly express their true feelings. The ability to identify subtle evidence of psychosocial health issues therefore largely depends on the experience of the assessors. These limitations affect both the efficiency and accuracy of the psychosocial health assessments.

In recent years, natural language processing (NLP) techniques^21–23^ are used to detect mental illness including depression, suicide, stress, anorexia, etc. Both traditional machine learning and deep learning methods are employed although deep learning methods receive more attention and perform better. However, these research works mainly study social media posts such as Twitter, Facebook, Reddit, etc. These may miss non-netizens such as elderly people who seldom share their thoughts through social media platforms. Other than that, the learning methods may not provide possible reasons for the causes or explanatory factors of mental illness. The methods, especially most of the deep learning techniques are treated as black-box methods that are hard to know why the methods work well. The causes are important for clinicians that can guide them to mental health diagnosis.

In the face of the tremendous surge in demand for psychosocial services, we developed an automatic speech analytics program (ASAP) for the detection of psychosocial health issues based on clients’ speech. ASAP is a digital health technology that involves a novel use of topic modeling, which is a technique used in machine learning. Topic modeling can discover the abstract “topic” in human speech by analyzing the words said. As the theory on linguistic behavior demonstrates, our verbal communication contains both conscious and unconscious components^24^. “What we say” in a conversation is the conscious component, containing messages that we intend to convey. Meanwhile, “how we say” something in a conversation is the unconscious component, reflecting our psychosocial status that we do not intend to reveal^25^. Owing to this behavior, when people are talking about certain topics, they will unconsciously include certain keywords in their speech^26^; for example, when people are upset, their speech will contain more negative words. Accordingly, identifying keywords in clients’ speech gives us a better chance of assessing their psychosocial status more accurately. Previous studies indicated that analyzing the frequency of word count in spoken text can be an indicator of various psychosocial disorders, including depression^27^, generalized anxiety disorder^28^, and schizophrenia^29^. Moreover, the ASAP can efficiently obtain adequate data for analysis by asking clients only a few questions. It therefore reduces the time required for an interview and increases the efficiency of the assessment. Furthermore, the automated program allows for cost-effective assessments as well as making psychosocial health care services more accessible. In this study, we successfully distinguish family caregivers with either high or low stress levels through analyzing their speech using ASAP, thus demonstrating the feasibility of providing easily accessible, efficient, accurate, and cost-effective psychosocial health care services using digital health technologies.

## Methods

### ASAP development

**Fig. 1:**
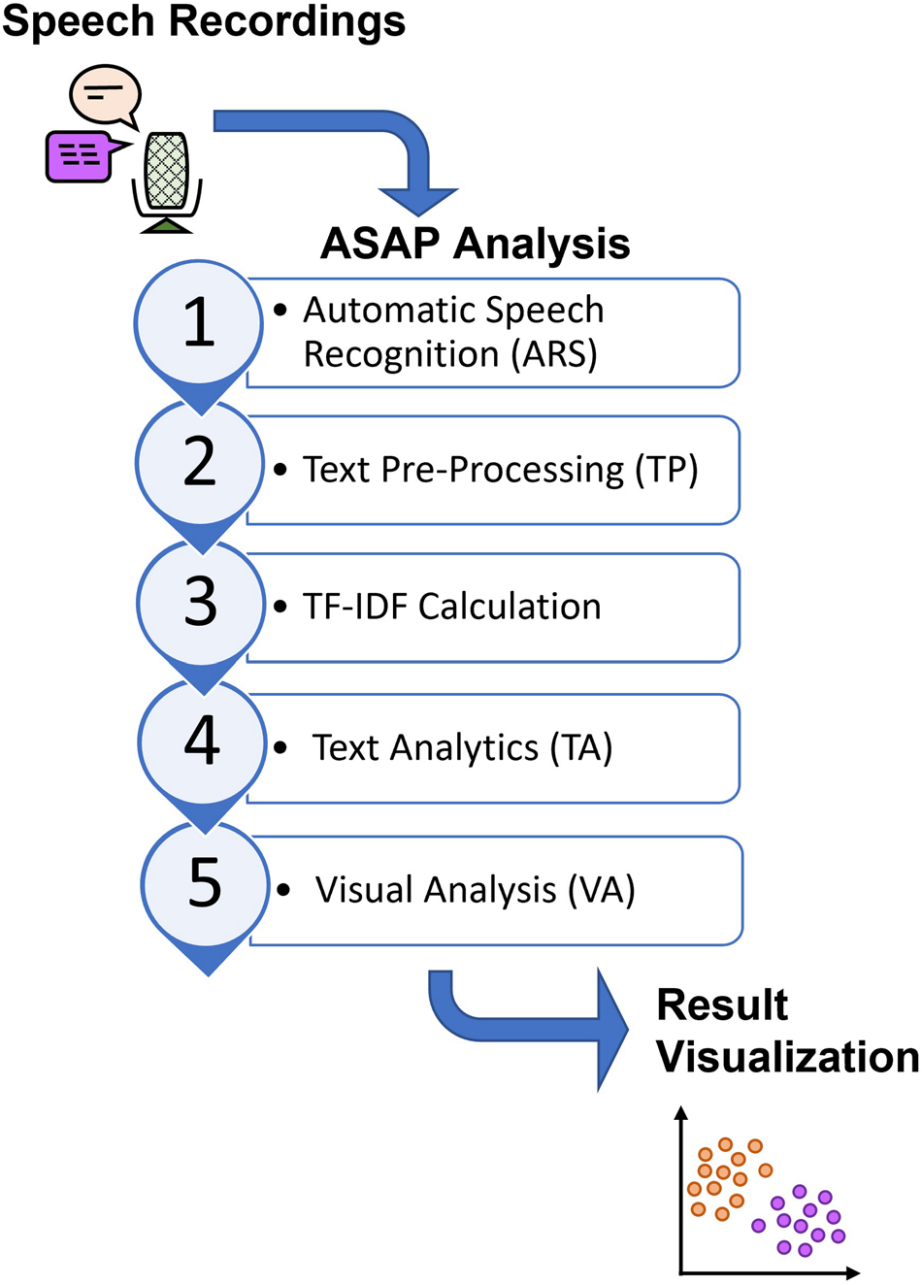
ASAP workflow diagram. ASAP contains five parts, which are (i) automatic speech recognition (ASR), (ii) text pre-processing (TP), (iii) TF-IDF calculation, (iv) text analytics (TA), and (v) visual analysis (VA). Speech recordings of caregivers were analyzed by ASAP and the clustering results were generated automatically.

ASAP was developed to perform a stress burden assessment using speech analysis. The workflow of ASAP is shown in Fig. 1. The system contains five parts, namely, automated speech recognition (ASR), Text Pre-processing (TP), term frequency–inverse document frequency (TF–IDF) calculation, text analytics (TA), and visual analysis (VA).

### ASAP part 1: Automated speech recognition (ASR)

We converted the interview audio tracks into the same file format (for example, wav). Then, we used the Google Cloud Speech API service to perform the speech to text processes to obtain the audio transcript for text analysis^30^. ASR allows for the automatic conversion of speech into text. The system can also separate the speech signal into chunks to be grouped into different word tokens^31,32^. To ensure the entire accuracy of the conversion, we performed common Chinese or Cantonese data cleansing before passing the transcripts into further analysis, such as removing punctuations and whitespaces^33^. Following that, the text was further analyzed using TP.

### ASAP part 2: Text Pre-processing (TP)

Cantonese is the spoken language of the family caregivers and is very different from written Chinese such as Traditional Chinese. It consists of foreign terminology and has a large set of Hong Kong specific terms. This makes the language analysis challenging. Common linguistic tools such as Linguistic Inquiry and Word Count are not applicable to analyze the textual form of Cantonese. Therefore, we adopted a recently developed Python package, PyCantonese, to analyze the textual form of Cantonese. PyCantonese is a Python library for Cantonese linguistics and NLP. However, it has limited accuracy to part-of-speech tagging and sentence parsing. This leads us to employ only two of its functions, including word segmentation and stop words processing. Cantonese is different from English in that Cantonese does not use a space between two words—for example, “解決問題” means “solving problems”. Cantonese also contains numerous word segments, which carry different meanings from the words constituting them—for example, “家人” (family members) is different from “家” (family) and “人” (people). We applied word segmentation to divide the text into meaningful words and word segments using the Python package, PyCantonese. Thereafter, we obtained a set of unique word tokens, though it may contain different languages (for example, English, Chinese, or Cantonese), symbols (including punctuation), and some spoken numbers. Given that our aim was to analyze the content based on the frequency of meaningful word tokens in each document, frequently shown symbols, numbers, and stopwords were not necessary. Thus, we removed them by matching them to the stopwords library (PyCantonese) to clean up the text. After the word segmentation, a list of unique word tokens was identified for subsequent TF–IDF analysis.

### ASAP part 3: Term frequency–inverse document frequency (TF–IDF) calculation

TF–IDF calculation can eliminate both common and rare words, which contain minimal information for differentiating caregivers’ stress burden levels^34^. TF–IDF measures the importance of a word in a document and consists of two parts: term frequency (TF) and inverse document frequency (IDF). TF measures the frequency of a word in a document, whereas IDF measures the frequency of a word across all the documents. Intuitively, a word that appears frequently in a document demonstrates its importance. However, this situation is often not the case if the word also appears frequently in all the other documents. For example, the word “only” appears frequently in all the documents in our data. Evidently, this word does not contain any discriminative information for identifying a caregiver’s stress burden level. To eliminate these types of common words, IDF counts the word frequency across all documents and weighs the contributions of these words. Given that TF counts the occurrence of words, the combination of TF and IDF (that is, TF–IDF) can discard words that appear frequently in all documents and retain words that appear frequently in a document. Not only common words, but rare words also contain minimal information. For example, the word “WhatsApp” only appeared in a few documents and did not appear in other documents in our data; that is, this word does not contain discriminative information and should be discarded as well. We introduced rules to eliminate common and rare words, including (i) the total weights of the TF–IDF of a word had to be at least 0.5 and (ii) the ratio between the maximum weight and the total weights of the TF–IDF of a word had to be at least 0.1. Similar rules are adopted to filter out less discriminative information^35^. The total weights of the TF–IDF of a word measures the total contributions of a word across all the documents. A small value implies that the occurrence of this word is low in all documents and therefore, it is potentially a rare word and should be discarded. The second rule is designed to eliminate common words; if a word commonly appears in most documents, then the TF–IDF weights of this word are similar across different documents. These similarities can be measured using the ratio between the maximum weight and the total weights of the TF–IDF of that word. A small value implies that this word is a common word. After performing the TF–IDF analysis, a list of meaningful words that do not include common or rare words are ready for TA analysis.

Other than TF-IDF, word embeddings^36^ and n-gram^37^ are popular features in text analysis. Word embeddings represent words using numerical vectors that put similar words close to each other. However, this approach requires many samples to obtain an accurate estimation. N-gram constructs a sequence of n nearby words. However, as different combinations of nearby Cantonese characters can have very different meanings, n-gram may create words that may be difficult to understand.

### ASAP part 4: Text analytics (TA)

**Fig. 2:**
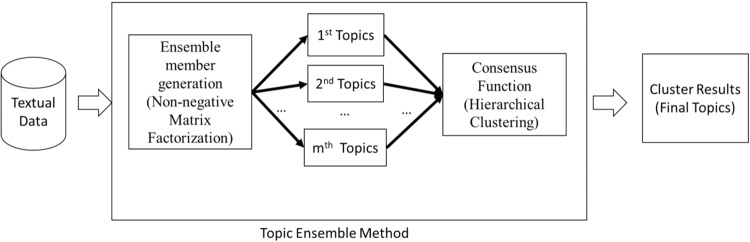
Illustration of the proposed ensemble modeling. In phase 1, Non-negative Matrix Factorization (NMF)^58^, a popular topic modeling tool, is applied to discover 10 different topics of the documents. Similar to other topic modeling tools, NMF generates different topics with different random number generators. To remove the random effect, we apply NMF with 70 different random number seeds. This produces 70 different sets of topics. Each set has 10 topics. These 70 × 10 topics are the ensemble members. In phase 2, the hierarchical clustering (HC)^59^ with complete linkage is adopted to integrate the ensemble members obtained in phase 1 and form two clusters. The HC is a clustering method that groups similar samples together. In other words, the HC identifies two groups by combining 700 topics.

TA can analyze the text and discover the abstract “topic” in human speech using the topic ensemble method. The ensemble method^38–42^ combines multiple learning algorithms to analyze complicated datasets and achieve a better performance than a single learning algorithm. It has been successfully applied in a wide range of classification tasks and clustering problems, including facial recognition^43^, object recognition^43^, and speech recognition^44^. The proposed ensemble method consists of two phases and they are (1) Ensemble Member Generation; and (2) Consensus Function. More explanations can be found in Fig. 2.

### ASAP part 5: Visual analysis (VA)

We used a symmetric diagram to display the two clusters, which were obtained by the proposed ensemble method, based on the analysis of the keywords that appeared in the respondents’ speech and then used multi-dimensional scaling to visualize the clustering results. We also listed the top ten keywords of each topic from caregivers with low and high stress burden levels in the current study.

### ASAP application on stress burden analysis: study design

An ASAP has been applied to analyze the speech of family caregivers. Eligible family caregivers were asked 12 open-ended questions regarding their families (Table 1). An ASAP analysis identified family caregivers with a different stress burden.

**Table 1:** Interview questions related to Walsh’s family resilience theory.

Walsh’s Theory (Three broad processes)	Interview Questions
Family Belief System	1. What make you stressful?
	2. When you feel bad, what you will do to make yourself feel better?
	3. What does it mean by adversity according to your experience?
	4. What keeps you persevering in the face of adversity?
Organizational Pattern	5. Who is on your mind when you feel weak and why?
	6. How many new friends did you make at the Center^a^? (The HKSKH Lady MacLehose Center)
	7. Do you think making new friends are helpful to you?
	8. Is there anything make you feel deeply at the Center?
Communicational Pattern	9. What troubles you the most when taking care of your family members?
	10. Who do you talk to when you are unhappy and why?
	11. Who in your family motivates you the most and why?
	12. What gives you the most satisfaction in caring for your family members?

^a^the participating non-profit organization providing integrated family and community services in this study.

Some 100 Cantonese-speaking family caregivers were recruited for the study at a non-profit organization (HKSKH Lady MacLehose Center) that provides integrated family and community services in Hong Kong. Potential participants were approached and screened for caregiver stress burden through social workers’ professional judgment and were then verified by the Caregiver Burden Inventory (CBI)^45,46^. Before interviewing the potential participants, the social workers explained the study and obtained signed informed consent from the family caregivers. All the family caregivers took part on a voluntary basis. This project was approved by the Human and Artefacts Research Ethics Committee of The Hong Kong University of Science and Technology (Protocol no.: HREP-2021-0213).

The CBI is a 24-item self-report scale that evaluates caregiver burden through a multi-dimensional approach^45^. It consists of five subscales, including time-dependence, developmental, physical, social, and emotional burden. A validated Chinese version of the CBI was adopted in this study. The Cronbach’s alpha of the Chinese CBI was 0.95^47^. Participants in this study were asked to assess each item on the Chinese CBI using a five-point Likert scale, where 0 is not at all descriptive and 4 is very descriptive. A higher score indicates a greater caregiver burden. A total score of 36 or lower indicates that low levels of stress are present while a score that totals over 36 indicates a high degree of stress^46^.

Finally, 47 caregivers with high stress levels and 53 caregivers with low stress levels were recruited on a voluntary basis. A total of 100 family caregivers were asked 12 interview questions about their family conditions and their responses were recorded for the ASAP analysis.

### ASAP application on stress burden analysis: Interview questions design

Twelve general questions regarding their family and family resilience were designed for the interview and are listed in Table 1. The 12 questions cover the three broad processes of Walsh’s family resilience theory: family belief systems, organizational pattern, and communication pattern. Family belief systems indicate the ability to overcome a crisis through making meaning in the face of adversity, maintaining a positive outlook, and maintaining spiritual belief. Organizational pattern indicates the presence of supportive family relationships that are flexible, connected, and have easy access to social networks and economic resources. Communication pattern indicates the capacity of family members to communicate effectively using communication skills that ensure clarity, allow for open emotional expression, and facilitate problem-solving in the face of adversity^48^.

According to our previous studies, family resilience is closely related to caregiver stress burden where high family resilience is correlated with low stress levels. Asking open-ended questions related to family resilience is a less sensitive topic than asking questions about caregiver stress. These questions allow participants to casually talk about their daily life and express their true feelings. Since family resilience is strongly correlated with stress levels, these non-sensitive questions can reflect the stress burden of the participants. The 100 eligible caregivers were asked to answer all 12 questions and their answers were recorded for subsequent analysis.

## Results

### Clustering results

**Fig. 3:**
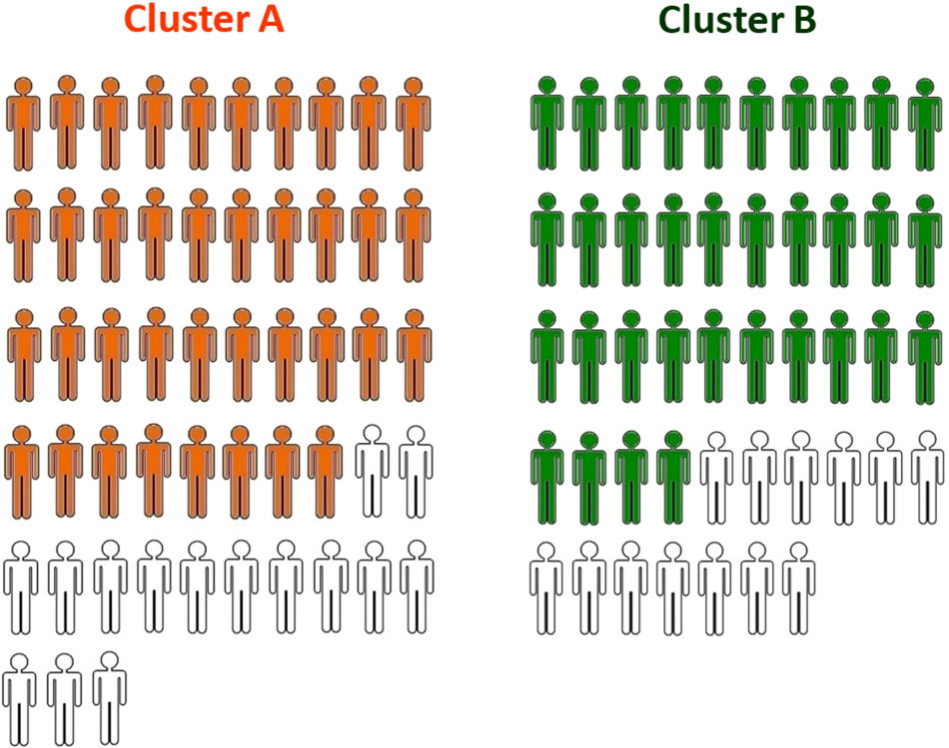
Symmetric diagram of ASAP analysis result. According to ASAP analysis, the 100 caregivers can be grouped into cluster A (orange) and cluster B (green) based on the types and frequency of keywords appeared in their speech. Cluster A contains 38 of the 53 low-stress caregivers while cluster B contains 34 of the 47 high-stress caregivers.

**Fig. 4:**
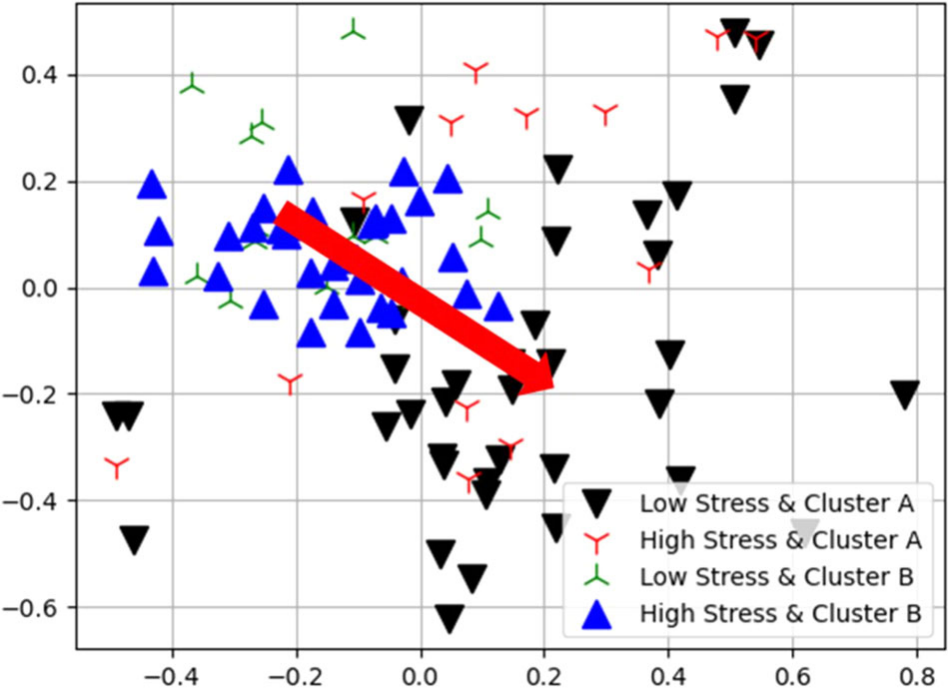
Visualization of the clustering results using multi-dimensional scaling (MDS). The blue up and dark down triangles represent the caregivers with low stress that belongs to cluster A and high stress that belongs to cluster B, respectively. We can see that these two groups are separated apart. The red arrow indicates the discriminative direction that differentiates the two groups of caregivers.

The results of this study indicated that the ASAP could distinguish between family caregivers with high or low stress burden levels. According to the ASAP analysis, caregivers could be grouped into two clusters—that is, Clusters A or B—based on the types and frequency of keywords in their speech. Cluster A contains 38 of the 53 caregivers with low stress levels while cluster B contains 34 of the 47 caregivers with high stress levels (Fig. 3 and Table 2). The results indicated that the accuracy rate of distinguishing between caregivers with high or low stress levels is 72% (that is, (38 + 34)/100 × 100% = 72%). Figure 4 visualizes the clustering results of the proposed method using multi-dimensional scaling. It further indicated a strong correlation between caregivers with low levels of stress and cluster A as well as between caregivers with high levels of stress and cluster B.

**Table 2:** Confusion Matrix of the Proposed Method.

	Low-stress burden	High-stress burden
Cluster A	38	13
Cluster B	15	34

### Performance assessment

We compare the performance of the proposed unsupervised learning method in ASAP with five supervised learning methods. The five methods are three versions of support vector machine (SVM)^49^ and two deep learning methods. The kernels adopted in SVM are linear kernel (SVM (Lin)), radial basis function kernel (SVM (Rbf)), and sigmoid function kernel (SVM (Sig)). The two deep learning methods are word embedding (WE)^50^ approach and recurrent neural network (RNN)^51^ approach. For the supervised learning methods, we use 10-fold cross validation technique to validate their performance. As there are 100 documents in total, this implies there are 90 documents for training and 10 documents for testing. The accuracy rate, true positive (low-stress) rate and true negative (high-stress) rate are reported in Table 3. The accuracy of the proposed method is the highest. It is better than the second-best method (SVM (Rbf)) over 10%. Besides, the ratio of true positive (low-stress) rate and true negative (high-stress) rate of the proposed method is close to one (71.70/72.34 $\approx$ 1). This balanced ratio implies that the proposed method is not biased to any one of the two classes. The performance to classify high-stress and low-stress are above 70%. However, SVM and Deep Learning methods are generally biased to the true positive (low-stress) rate. The true negative (high-stress) of SVM (Lin) and Deep Learning (WE) are even close to zero. This means they are not able to classify the high-stress cases. The following provides some possible reasons. The proposed method adopts the topic modeling tool, Non-negative Matrix Factorization, to identify words to form topics. Different topics may consist of different words of the documents. In other words, not all the words are used in the classification. However, for the three versions of SVM, they use all the words for classification. As some of the words may be noise, they can confuse SVM and limit their applicability to this data. This may be the reason why the proposed method performs better than SVM. The proposed method is also better than the two deep learning methods. The deep learning methods require a large sample size to generate good results. However, in this study, there are only 90 documents for training. This may not be enough for deep learning to train up a high-performance classifier.

**Table 3:** Performance Comparison (in Percentage) Using Different Machine Learning Methods.

	True Positive (low-stress) Rate (%)	True Negative (high-stress) Rate (%)	Accuracy Rate (%)
SVM (Lin)	100.00	2.00	54.00
SVM (Rbf)	79.71	41.08	61.00
SVM (Sig)	60.86	54.76	59.00
Deep Learning (WE)	100.00	0.00	53.00
Deep Learning (RNN)	71.24	43.19	56.00
Proposed Method	71.70	72.34	72.00

We study the impacts of the number of words toward the performance of the proposed method. We rank all the words using mutual information score^52^. The score measures the similarity between the TF-IDF features of words and the class information (i.e., low-stress or high-stress). A larger score implies a higher association with the class information and thus implies the feature is more important. We select the top 10%, 20%, …, 90%, 100% of words as an input to the proposed method. The accuracy rates are shown in Table 4. The highest accuracy rate is reached at 60%. The accuracy rate is then dropped and go back to 72. The results imply that some words are not relevant and confuse the proposed method. Those words may be from interview questions that are less related to the stress level. One possible question is the interview question 8 in Table 1. Both low-stress caregivers and high-stress caregivers can feel deeply at the Center.

**Table 4:** Percentage of words selected as an input for the proposed method.

Percentage of Words	10%	20%	30%	40%	50%	60%	70%	80%	90%	100%
Accuracy Rate	60	67	69	70	71	75	71	65	69	72

The keywords identified in the two clusters also demonstrated substantial differences (Table 5). More salient topics were identified for caregivers with a lower stress level (eight topics) than in caregivers with a higher stress level (three topics).

**Table 5:** Top 10 keywords of each topic of low-stress and high-stress burden caregivers.

Low-Stress Caregivers								High-Stress Caregivers		
**Topic 1**	**Topic 2**	**Topic 3**	**Topic 4**	**Topic 5**	**Topic 6**	**Topic 7**	**Topic 8**	**Topic 1**	**Topic 2**	**Topic 3**
多謝 (Thank you)	即時 (Immediate)	活動 (Activity)	交換 (Exchange)	阿女 (Daughter)	冇病冇痛 (No illness and pain)	旅行 (Travel)	阿仔 (Son)	媽媽 (Mother)	解決 (Resolve)	先生 (Husband)
旅行 (Travel)	成長 (Growth up)	阿仔 (Son)	電話 (Phone)	家姐 (Elder sister)	開開心心 (Happy)	多謝 (Thank you)	隨便 (casual or informal)	見下 (Meet)	方法 (Method)	阿女 (Daughter)
冇病冇痛 (No illness and pain)	溝通 (Communicate)	定係 (Or else)	邊一個 (Which one)	煮飯 (Cook)	解決 (Resolve)	辛苦 (Hard and exhausting)	旅程 (Journey)	行下 (Walk)	需要 (Need)	學校 (School)
開開心心 (Happy)	帶畀 (Bring to)	鍾意 (Like)	約出 (Appointment)	行街 (Wandering)	多謝 (Thank you)	裏面 (Inside)	籃球 (Basketball)	放假 (Vacation)	本身 (Self)	姑娘 (Nurse)
辛苦 (Hard and exhausting)	多謝 (Thank you)	希望 (Hope)	阿女 (Daughter)	關係 (Relationship)	動力 (propulsion)	學習 (Learn)	有得 (Have)	多數 (Majority)	處理 (Deal with)	繼續 (Continue)
裏面 (Inside)	諗起 (Think of)	平時 (Most of the time)?	健康 (Healthy)	出去 (Go out)	堅強 (Strong)	細路仔 (Kids)	狀態 (Condition)	出街 (Go out)	心情 (Mood)	出去 (Go out)
解決 (Resolve)	鍾意 (Like)	工作 (Work)	有少少 (A little bit)	改變 (Change)	話畀 (Tell)	朋友 (Friend)	點解會 (Why)	爸爸 (Father)	冇乜 (Not much)	家姐 (Elder sister)
動力 (propulsion)	快樂 (Happy)	咩嘢 (What up)	開朗 (Cheerful)	同人 (With others)	旅程 (Journey)	多數 (Majority)	定係 (Or else)	最緊要 (The most important)	最主要 (Mainly)	頭先 (A while ago)
學習 (Learn)	見面 (Meet up)	瞓覺 (Sleep)	當中 (In the middle)	經常 (Frequently)	嚟自 (Come from)	感覺 (Feeling)	平時 (Most of the time)	其他人 (The others)	媽媽 (Mother)	少少 (A little bit)
過嚟 (Come here)	另外 (In addition)	興趣 (Interest)	希望 (Hope)	脾氣 (Temper)	需要 (Required or needed)	大概 (Probably)	即刻 (Immediate)	今日 (Today)	地方 (Place)	以前 (In the past)

## Discussion

The digital health program, ASAP, was successfully developed in this research study. The ASAP can distinguish between family caregivers with high or low stress levels by analyzing their speech. These research findings indicate the potential of using digital health technology to assist in psychosocial health assessments. ASAP can provide an accurate, efficient, and cost-effective initial assessment to identify higher stress burden among family caregivers. The information can be referred to social workers and healthcare professionals for follow-up actions.

The ASAP can identify family caregivers with a low or high stress burden with high accuracy. According to the results of this study, the accuracy for the ASAP to distinguish between a low or high stress burden is 72%. The accuracy is the highest when we compared the performance of the proposed unsupervised learning method in ASAP with five supervised learning methods, including SVM(Lin), SVM (Rbf), SVM (Sig), WE, and RNN. Identifying stress burden by analyzing people’s verbal responses to non-sensitive questions can greatly reduce the biases in psychosocial health assessments. Answering non-sensitive questions allows people to casually talk about themselves and express their true feelings. It can help to reduce bias since due to the stigma of psychosocial health problems, people will be more willing to openly express themselves if the questions are not directly concerned with these problems. Moreover, the digital health program can analyze data based on a technique from machine learning, which may provide more objective and consistent assessments.

Our results in Table 5 found that more salient topics were identified for family caregivers with a low stress burden than those with high stress burden. One possible reason is that caregivers with a lower stress level are more willing to share their experiences and exchange information with others, whereas caregivers with a higher stress level are less willing. This is also reflected in Fig. 4. We can observe in Fig. 4 that the distribution of the low-stress burden caregivers was more spread out than that of the high-stress burden caregivers. Table 5 shows that the low-stress burden caregivers shared more relaxing issues such as “Travel”, “Interest”, “Appointment”, “Wandering”, while the high-stress caregivers mainly shared issues about their family members and the words they always used included “Mother”, “Father”, and “Husband”. By reviewing the interview scripts, we found that one of the common stressors of the high-stress burden caregivers was from their family members, with whom they may not be satisfied with their relationship. On the contrary, the low-stress burden caregivers expressed more gratitude than those with high stress burden. Among the eight topics of the low-stress burden caregivers, “happy” appeared three times and “thank you” appeared four times. However, these words did not appear in the high-stress burden caregivers. This echoes the psychology that people who are more stressful are generally less likely to be happy. These results indicated that the proposed ASAP is feasible to assess the caregivers’ stress burden.

The efficiency of the psychosocial health assessment can be improved by using the ASAP. The analysis was based on participants’ answers to 12 questions regarding their family. The average interview time was around 30 minutes, which is shorter than the average time of a conventional assessment that requires 1 to 2 hours to complete several validated instruments. The ASAP therefore demonstrates the potential to provide highly efficient psychosocial health assessments.

ASAP is an automated digital health program. Together with a high accuracy rate and efficiency, ASAP has the potential to greatly improve the accessibility of psychosocial health care services. Limited health care resources reduce access and is one of the major problems of psychosocial health care services. It was reported that only 10% of people who require psychosocial health care services can access the care they need in low- and middle-income countries^18^. The ASAP can speed up the initial psychosocial health assessments and provide cost-effective psychosocial health screenings to many people. For those who were identified to have psychosocial health issues, they can be referred to health care professionals for further assessments and treatments. The ASAP is therefore a fully scalable system for assessing a wide variety of psychosocial problems in the future. It has the potential to assess different psychosocial problems, including acute panic, anxiety, obsessive behaviors, hoarding, paranoia, and depression. It can also greatly increase the accessibility of psychosocial support services. The ASAP, therefore, has the potential to be an effective tool for society to deal with the ever-growing psychosocial health crisis.

This work also provides a showcase for analyzing text in non-dominant languages and is applicable to other languages. The language we analyzed was Cantonese, which is the language of the family caregivers. However, few NLP tools were available. This made our analysis difficult because the text contained a lot of irrelevant information. In English, many Python language tools can use part-of-speech tagging and word association to extract relevant information. Without the help of these tools, we adopted TF-IDF calculation and introduced a set of rules to filter out less discriminant information. These techniques are applicable to other languages and particularly useful to languages with few analysis tools.

Further research is needed to improve the use of the ASAP in psychosocial health assessments. Firstly, the program can be modified to further improve the accuracy in distinguishing family caregivers with high or low stress burden. Secondly, the number of questions and the content of each question can be modified to further improve the efficiency of the digital psychosocial health assessment. Thirdly, the ASAP can be further developed for the auto-detection of a wide range of psychosocial health issues, including acute panic, anxiety, obsessive behaviors, depression, social phobia, and communication disorders. Fourthly, similar to other AI chatbots^53–55^ in the field of mental health, the ASAP can also empower large language models (LLM) such as ChatGPT or GPT-4 for mental illness detection. The interview questions and topic modeling results discussed in this paper can serve as a foundation for the detection of the mental illness and the interpretation of the detection results.

In the current study, we employed a clear cut-off for the CBI of either side of 36 to indicate low level of stress (a total score of 36 or lower) and high level of stress (a total score over 36). This may affect the classification accuracy. Further evidence is needed to support the cut-off and the classification accuracy. There are also some limitations that may impede the applications of ASAP. First, there is little evidence that digital health program can be successfully incorporated into healthcare system^56^. Although the usefulness of digital programs has been demonstrated in research studies, few reports have confirmed their usage in clinical settings. One problem is that research has assumed the technology itself can be a stand-alone service, while paying less attention to the ecosystem in clinical settings, such as human support and organizational factors^56^. Our research overcomes this obstacle by designing and developing ASAP as a user-friendly tool for healthcare professionals. It can assist healthcare professionals by streamlining the health assessment process and therefore can be easily integrated into the healthcare system to improve the efficiency of healthcare services. Second, technological barriers can limit the accessibility of digital health programs^57^. Most digital services required the clients to have devices with internet connectivity to access the services. In our research design, it is not necessary for the clients to possess special devices. ASAP can perform health assessment whenever the clients can make an ordinary phone call or come to the healthcare institutes in-person.

With practical measures to minimize the limitations of digital health services, the development of ASAP has a great potential to promote psychosocial health. With the long-lasting effects of COVID-19 and increasing number of global conflicts, the healthcare system of societies and the psychosocial health of people are in jeopardy around the world. Improving the use of digital health technologies in healthcare services is a potential way to handle the surge in cases of psychosocial health problems. It is also critical for ensuring the productivity of societies for their future development.

## Data Availability

Aggregated data that support the findings of this study may be available upon request by contacting the corresponding author, Prof. Mike K. P. So. Any request for data will be evaluated and responded to in a manner consistent with policies intended to protect participant confidentiality and language in the study protocol and informed consent form.

## Code availability

Code for all analyses was written in Python 3.9. This code may be available upon request by contacting the corresponding author, M.K.P.S. Any request for Python scripts will be evaluated and responded to in a manner consistent with policies intended to protect participant confidentiality and language in the study protocol and informed consent form.
